# Heart rate variability and the relationship between trauma exposure age, and psychopathology in a post-conflict setting

**DOI:** 10.1186/s12888-016-0850-5

**Published:** 2016-05-10

**Authors:** Belinda J. Liddell, Andrew H. Kemp, Zachary Steel, Angela Nickerson, Richard A. Bryant, Natalino Tam, Alvin Kuowei Tay, Derrick Silove

**Affiliations:** The Psychiatry Research and Teaching Unit (PRTU), School of Psychiatry, University of New South Wales, Sydney, Australia; School of Psychology, University of New South Wales, Sydney, NSW 2052 Australia; Discipline of Psychiatry, University of Sydney, Sydney, NSW Australia; St John of God, Richmond Hospital, North Richmond, NSW 2754 Australia; Black Dog Institute, School of Psychiatry, University of New South Wales, NSW Sydney, Australia; Academic Mental Health Unit, South Western Sydney Local Health District, Sydney, Australia

**Keywords:** Heart rate variability (HRV), Posttraumatic stress disorder, Potentially traumatic event (PTE), Trauma, Aggression, Depression, Age, Post-conflict, Timor-Leste, East Timor

## Abstract

**Background:**

Cumulative exposure to potentially traumatic events (PTEs) increases risk for mental distress in conflict-affected settings, but the psychophysiological mechanisms that mediate this dose-response relationship are unknown. We investigated diminished heart rate variability (HRV) – an index of vagus nerve function and a robust predictor of emotion regulation capacity - as a vulnerability marker that potentially mediates the association between PTE exposure, age and symptoms of posttraumatic stress disorder (PTSD), psychological distress and aggressive behavior, in a community sample from Timor-Leste — a post-conflict country with a history of mass violence.

**Method:**

Resting state heart rate data was recorded from 45 cases of PTSD, depression and intermittent explosive disorder (IED); and 29 non-case controls.

**Results:**

Resting HRV was significantly reduced in the combined case group compared with non-cases (*p* = .021; Cohen’s *d* = 0.5). A significant mediation effect was also observed, whereby a sequence of increased age, reduced HRV and elevated PTSD symptoms mediated the association between PTE exposure and distress (*B* = .06, *SE* = .05, 95 % CI = [.00–.217]) and aggression (*B* = .02, *SE* = .02, 95 % CI = [.0003–.069])).

**Conclusion:**

The findings demonstrate an association between diminished resting HRV and psychopathology. Moreover, age-related HRV reductions emerged as a potential psychophysiological mechanism that underlies enhanced vulnerability to distress and aggression following cumulative PTE exposure.

## Background

Exposure to potentially traumatic events (PTEs) is known to increase risk for mental disorder [1, 2]. Cumulative exposure to PTEs in conflict-affected communities, including mass violence and human-rights abuses, is a strong predictor of PTSD, depression [1, 3, 4] and anger/Intermittent Explosive Disorder (IED) [5] - an association that is known as the dose-effect relationship [2]. This association has been attributed to a sensitization mechanism in which exposure to earlier events increase vulnerability to subsequent PTEs, resulting in enhanced risk for further psychopathology [6]. Longitudinal studies amongst veteran and other trauma-exposed populations have also shown that posttraumatic stress symptoms following PTE exposure may precede and contribute to the onset of depression and other anxiety disorders over time [7–9]. To date, there has been limited investigation of these relationships in post-conflict settings where populations have had prolonged exposure to extreme trauma. Furthermore, there has also been no study of the psychophysiological mechanisms that may underpin this increased sensitivity to psychological distress following cumulative PTE exposure in such settings.

One potential psychophysiological marker of increased sensitivity to a range of affective responses following trauma is reduced heart rate variability (HRV), reflecting altered vagal nerve function [10] and impaired emotion regulation capacity [11]. HRV indexes parasympathetic regulation of heart rate via the inhibitory influence of the myelinated vagus nerve over the sinoatrial node [10, 12]. As such, under conditions of safety, heart rate is slowed, and social engagement is facilitated [10, 13]. In response to a salient external cue – such as a threat signal – the vagal brake is released, allowing the sympathetic nervous system to dominate and mobilize defense responses [10]. Exposure to chronic threat in a conflict-affected context may affect the functioning of the vagal system in the long term, impairing adaptive reactions to stressful events [14].

A higher level of resting-state HRV has been postulated to reflect a central indicator of healthy emotion regulation capacity [11, 15], indicating a system that is able to effectively respond to environmental challenges. Accordingly, a healthy vagal system is thought to index psychological flexibility, emotional self-regulation and positive adaptation [10, 12]. Conversely, low resting state HRV is an indicator of psychophysiological rigidity characterized by a diminished capacity for regulating emotional responses to distressing events [11]. Reduced resting HRV has been associated with greater trauma exposure [16], mental disorder and symptoms, and physical health status [17], including PTSD [18]; depression and anxiety [19–21]; aggression and anger [22]; comorbidity [19, 20], and poor physical health [17]. Studies have also linked reduced resting HRV with increased vulnerability to distress [16] and delayed physiological recovery following stress exposure [23]. As yet, however, little is known about the role of HRV in mediating the relationship between cumulative exposure to PTEs and common forms of mental disorder.

HRV also decreases naturally with advancing age [24–27]. There is evidence that key HRV measures indexing parasympathetic influences on the heart decrease by 1.4 % with every advancing year in a healthy sample aged 50–72 years, after controlling for gender and lifestyle factors (e.g., smoking, alcohol consumption) [26]. Commentators have suggested that this pattern reflects reduced autonomic responsivity to the external environment with older age, a pattern that could be modified through physical exercise and weight loss [24]. It is therefore important to account for age influences over HRV when examining its association with mental health factors.

The present study examined these issues in Timor-Leste, a population exposed to repeated PTEs related to mass conflict. Timor-Leste is a half-island country situated between Australia and Indonesia that has experienced decades of political violence, deprivation and insecurity. The Indonesian occupation of the country between 1975 and 1999 was characterized by widespread intimidation and oppression, with documented incidents of torture, disappearances and imprisonments [28, 29]. Following a United Nations facilitated referendum in 1999, Indonesian-supported militia initiated a campaign of violence leading to human rights violations, mass population displacement, and property destruction. Since independence in 2002, Timor-Leste has continued to experience periods of violence, most notably in 2006, with extensive displacement and infrastructure destruction following civil unrest. Whilst there is some progress in terms of socioeconomic development, conditions of poverty persist resulting in food insecurity, limited access to basic services and high unemployment [30].

A 6-year longitudinal mental health survey was conducted with Timorese community members in 2004 [31], and wave two data was collected in 2010–2011 [4]. Rates of PTSD and depression were found to increase across this period, attributable to exposure to human rights traumas, ongoing stress, and persistent perceptions of injustice, suggesting that a recurrent episode of violence as experienced in 2006 in Timor-Leste can have a major impact on mental health [4, 32]. Moreover, the prevalence of explosive anger was found to be particularly high in the Timorese community (38 %) [5]. Anger may reflect a persistent mental health concern, reflecting a sequential reaction to significant exposure to human rights violations followed by an adverse post-conflict environment [5, 33]. IED prevalence has been observed to be high in other post-conflict settings (e.g., South Africa [34]), and has been associated with psychiatric comorbidity and elevated functional impairment (e.g., in Iraq [35]). When considering mental health outcomes in Timor-Leste, it is therefore critical to include an assessment of pathological anger [36].

In the current study, it was hypothesized that resting HRV would be lower in a clinical case group comprising Timorese with PTSD, depression or IED, compared to a non-case group with no mental disorder. We were specifically interested in PTSD and psychological distress as common post-conflict mental health outcomes, but also IED due to the high prevalence of IED and anger in the Timorese population. Acknowledging the cross-sectional nature of the study, we examine a theoretical model to test whether age, HRV and PTSD symptoms play an intermediary role linking cumulative PTE exposure and mental health outcomes. In baseline models, we first explored the extent to which HRV was a direct mediator of PTE exposure on severity of PTSD symptoms, psychological distress and aggressive behavior, whilst accounting for age effects on HRV [24]. Drawing on evidence that PTSD symptoms may play an instrumental role in the development of other secondary forms of distress and anxiety [7–9], we also explored the extent to which reduced HRV *and* elevated PTSD symptoms in sequence mediate the impact of PTEs on distress and aggressive behavior symptoms [37].

## Methods

### Participants

Participants were recruited from a large community sample (>3000 people) participating in a mental health survey in Timor-Leste undertaken between May 2010 and June 2011 [4]. The subsample was primarily recruited for a clinical concordance study to establish convergence between measures implemented in the mental health survey and gold-standard diagnostic psychiatric clinical interviews [4, 36]. The concordance study followed a double-blind randomized probability case and non-case recruitment strategy, with an enriched case sample of probable PTSD, IED and major depressive episode (MDE), a procedure that ensures a balance of cases and non-cases in accordance with standard concordance methodologies [38]. The final sample comprised 97 participants from a pool of 189 individuals approached (overall response rate 51.4 %). Of these, 80 participants (29 males; 51 females) with a mean age of 40.4 years (SD =13.8) consented to participate in a heart rate assessment in accordance with ethics approval from the University of New South Wales Australia Human Research Ethics Committee.

### Diagnostic classification of PTSD, MDE, IED cases

Diagnostic status was determined by one of two clinical/research psychologists (BL, AT), who had experience and training conducting mental health assessments in conflict-affected populations (supervised by DS, ZS). Diagnoses were made according to the Structured Clinical Interview for DSM-IV (SCID-IV) [39] for PTSD, IED and major depressive episode. Non-cases were defined as not meeting the diagnostic criteria for PTSD, MDE, or IED. Interviews were conducted in Tetum and English with trained mental health Timorese interpreters.

Mental health measures were also undertaken as part of the validation of the community survey instruments (see Procedure below). PTSD symptoms were measured by the Harvard Trauma Questionnaire (HTQ), which has demonstrated cross-cultural validity [40], from which a continuous measure of PTSD symptom severity (HTQ score) was derived for use in the mediation analyses. PTE exposure was measured using the HTQ events scale reflecting conflict exposure, human-rights violations, witnessing murder, natural disaster and health stress [40], with minor additions suitable to the Timorese context. Trauma count was collapsed across the two time periods assessed in the longitudinal mental health survey (pre-2004 and post-2004) to reflect lifetime exposure [4].

Anger and IED symptoms were measured by a community questionnaire developed via extensive pilot testing and consultation to ensure cultural congruity [5, 36]. Responses on the three aggressive behavior (verbal, property destruction or physical) items were aggregated to compute an index of aggressive behavior frequency for application in the mediation analyses (Cronbach α = 0.73; scores ranged from 3–15). The K10 [41] was used to index a range of depression/anxiety symptoms, with scores reflecting general psychological distress.

### Procedure

Participants first completed a mental health assessment with a trained Timorese interviewer in which the HTQ, modified K10 and anger measure were administered. Following a break of at least 25 min, resting heart rate was recorded by the Polar RS800CX, a method validated against standard ECG measurement [42, 43] and widely used in experimental research [15, 44]. R-R interval data was sampled at 1000 Hz via a chest strap worn by participants that wirelessly sends signals to the RS800CX unit. Participants sat in a in a curtained, air-conditioned room, and were instructed to relax and breathe normally. Ten minutes of continuous recording commenced after a habituation period of 5 min. After lunch break of 30–45 minutes, participants then partook in a clinical interview with the Australian research psychologist.

### Data reduction and analysis

Heart rate data were analyzed in Kubios 2.0 (http://kubios.uef.fi). Following visual inspection, artefacts were manually corrected using the medium-strong level correction function. Six participants were excluded from further analysis due to uncorrectable noise in their heart rate data (*n* = 3); diagnosis of epilepsy (*n* = 1); and incomplete recording (*n* = 2). The final sample of 74 participants for analysis consisted of 45 cases of PTSD, MDE and/or IED and 29 non-cases (Table 1).

**Table 1:** Demographic data for case and noncase groups

	Cases		Noncases		
	n	%	n	%	χ2
Number	45		29		
Diagnoses (n)					
PTSD only	0	0.0 %			
IED only	26	57.8 %			
MDE only	4	8.9 %			
Comorbid PTSD and IED	2	4.4 %			
Comorbid PTSD and MDE	3	6.7 %			
Comorbid IED and MDE	2	4.4 %			
Comorbid PTSD, IED and MDE	8	17.8 %			
Sex (n)					<0.01
Males	17	37.8 %	11	37.9 %	
Females	28	62.2 %	18	62.1 %	
Marital status (%): Case group *n = 44* ^a^					0.08
Married	31	70.5 %	20	69.0 %	
Widowed	5	11.4 %	3	10.3 %	
Single/Never married	8	18.2 %	6	20.7 %	
Education (n): Case group *n = 44* ^a^					5.51
Completed university degree	2	4.5 %	3	10.3 %	
Completed secondary school	5	11.4 %	6	20.7 %	
Completed primary school	7	15.9 %	6	20.7 %	
Minimal formal education	30	68.2 %	14	48.3 %	
Employment (n): Case group *n = 44* ^a^					13.05**
Paid employment	11	25.0 %	12	41.4 %	
Unemployed	7	15.9 %	12	41.4 %	
Subsistence living or home duties	26	59.1 %	5	17.2 %	
Smoking history (n): Case group *n = 44* ^a^					1.27
Current	12	27.3 %	8	27.6 %	
Past smoker	4	9.1 %	5	17.2 %	
Never	28	63.6 %	16	55.2 %	

Chi-squared tests: *p < .05; ** *p* < .01

^a^Data relating to marital status, education, employment and smoking history missing from one clinical case

HRV was indexed by the root of the mean square of successive differences (RMSSD) in RR intervals. RMSSD is a stable variance-based index of mostly parasympathetic influences on heart rate over short term recordings [45]. RMSSD is a reliable measure of HRV when data is recorded in a non-laboratory environment that permits spontaneous breathing [46]. RMSSD data was log transformed due to significant kurtosis (skewness = 2.28, SE = .29; kurtosis = 6.33, SE = .57), and screened for outliers (+/− 2 standard deviations; constituting 4.4 % of data points). Outliers were replaced with the sample mean plus or minus 2 standard deviations.

#### Statistical analyses

To examine group differences, independent samples t-tests were conducted on HR/HRV, demographics and symptom measures (*p* < .05); and chi-square analyses were conducted on categorical data (*p* < .05). One-way ANOVAs with posthoc *t*-test contrasts were used to examine PTSD symptom differences between diagnostic categories (Bonferroni corrected). Bivariate correlations conducted in the full sample examined the inter-correlations between variables in preparation for mediation analyses (*p* < .007; Bonferroni corrected based on correlations tested between 7 variables). Cohen’s *d* was computed for HR/HRV group effects, with *d* > 0.5 indicating a moderate effect size [47].

A mediation model was constructed using a nonparametric bootstrapping mediation method by applying the PROCESS tool [37] in SPSS (version 21). Mediation modelling is a suitable analysis approach for understanding the directional relationships between variables within a cross-sectional dataset, allowing models based on theoretical assumptions to be tested [37]. We applied a multiple mediation model framework using conditional processing, allowing the analysis of direct and indirect mediating effects to test our hypotheses.

In a first set of baseline models, we investigated whether age-related HRV reductions mediated the impact of PTE exposure on each symptom domain, that is, PTSD, psychological distress and aggressive behavior. Given the age range of our sample (i.e., 19–68 years), we included age as a mediating factor in the model. This was to explicitly model the associations between age and both a) trauma exposure (older Timorese were more likely to be exposed to conflict-related trauma) and b) HRV based on a clear evidence-base of age-related HRV reductions [24–27]. Therefore, the key mediators were first age and second, resting HRV, with quantity of PTE exposure being the predictor variable. Sex was included as a covariate factor.

Second, a full mediation model was investigated that included PTSD symptoms as the third mediating variable, testing seriatim the effects of age-related HRV reductions and increased PTSD symptoms on the outcomes of psychological distress and aggressive behavior symptoms respectively. Alternative plausible models were also tested: a) psychological distress as the third mediator with PTSD as the outcome variable; b) aggressive behavior as the third mediator with PTSD as the outcome variable.

In all models, results were derived from 10,000 bootstrapped samples; unstandardized parameter estimates, standard errors and bias-corrected 95 % confidence intervals (95 % CI) determined the significance of direct and indirect (i.e., mediating) associations. Individual pathways were significant at *p* < .05.

## Results

### Case vs non-case group differences

Descriptive statistics by diagnostic status and inter-correlations are presented in Tables 1 and 2 respectively. Forty-six participants were female (62 %); the average age was 39.9 years (SD 13.4; range 19-68 years). The case and non-case groups did not differ on gender distribution (χ^2^ (1) = .000, *p* > .99); age (t (72) = 1.37, *p* = .18); marital status (χ^2^ (2) = .08, *p* = .96); education (χ^2^ (3) = 3.24, *p* = .34) or smoking history (χ^2^ (2) = 1.15, *p* = .56). Noncases were more likely to be in paid employment or seeking employment; whereas cases were more likely to be engaged in subsistence farming/home duties (χ^2^ (2) = 13.05, *p* = .001). The case group reported exposure to a higher number of lifetime PTEs (t(72) = 2.18, *p* = .032), reported greater severity of distress (higher K10 scores (t(72) = 3.39, *p* = .001)); PTSD symptoms (higher HTQ scores (t(72) = 6.97, *p*<. 001)); and levels of aggressive behaviour (t(72) = 7.02, *p* < .001) than the non-case group. Significant differences were observed between diagnostic categories and comorbid cases in regards to PTSD symptoms (F(1,6) = 31.52, *p* < .001). While PTSD cases demonstrated elevated PTSD symptom severity compared to non-PTSD (IED and MDE) cases ((t(67) = 5.38, *p* < .001), all diagnostic categories demonstrated a higher level of PTSD symptoms relative to non-cases (PTSD: t(67) = 10.16, *p* < .001; IED: t(67) = 9.93, *p* < .001; MDE: t(67) = 11.72, *p* < .001).

**Table 2:** Key demographic, symptom severity scores, comorbidity rates and heart rate variables presented on the left side of the table for case and noncase groups

		Cases		Noncases			Correlations						
		Mean	SD	Mean	SD	t	1	2	3	4	5	6	7
1	Age (years)	41.56	13.4	37.21	13.22	1.37	–						
2	PTE exposure (count)	5.44	2.37	4.21	2.40	2.18*	0.24	–					
3	K10 score (sum)	28.98	7.97	22.69	7.50	3.39**	0.27	0.23	–				
4	HTQ score (mean)	2.12	0.51	1.38	0.33	6.97**	0.15	0.38***	0.71***	–			
5	Aggressive behaviour index (score)	10.47	3.12	5.48	2.75	7.02**	−0.10	0.17	0.16	.50***	–		
6	HR (bpm)	75.18	12.68	77.84	11.47	−0.83	−0.15	–0.31***	−0.02	−0.07	–0.11	–	
7	RMSSD (ms; log)	1.46	0.26	1.58	0.22	−2.02*	−0.45***	−0.01	−0.23	−0.19	−0.04	−0.60***	–

*PTE* potentially traumatic event, *K10* 10 item measure of psychological distress symptoms with scores 25–29 indicating high levels of distress and scores > 30 extreme levels of distress, *HTQ* Harvard Trauma Questionnaire indexing PTSD symptoms, *HR* heart rate, *RMSSD* root of the mean square of successive differences HRV measure. Right side of table presents inter-correlations (case and non-case groups combined) between variables (*R*-values presented). For both independent samples *t*-test and bivariate correlations: * *p* < .05; **p<. 01; ***p<. 007 (Bonferroni-corrected for correlation analyses)

There was no significant difference between groups in resting heart rate HR (t(72) = −.91, *p* = .37), but HRV was significantly lower in the case group relative to the non-case group (t(72) = −2.07, *p* = .021 (1-tailed); Cohen’s *d* = 0.50), Table 2. Across the full sample, HRV was inversely correlated with HR and age (*r* values presented in Table 2). PTE exposure was negatively correlated with heart rate, but not with HRV. PTE exposure and PTSD symptoms were also positively correlated (see Table 2).

### Mediation analyses

#### Baseline mediation models

**Fig. 1:**
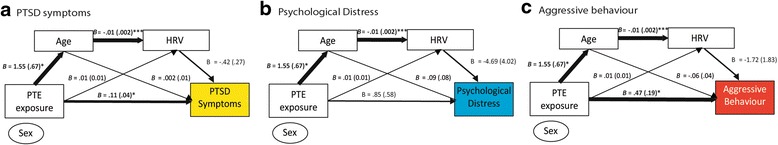
Mediation models testing the association between PTE exposure and **a** PTSD symptoms; **b** psychological distress symptoms and **c** aggressive behavior as a function of age and HRV. *Thick lines* indicate significant direct or indirect serial pathways. Effects sizes (standard errors) are presented for each path; * *p* < .05; ** *p* < .01; *** *p* < .001. The covariate of sex was not significantly associated with any of the variables in the model

The baseline models presented in Fig. 1 identified significant direct pathways between PTE exposure and both PTSD symptoms (*B* = .11, *SE* = .04, p = .014, 95 % CI = [.02–.19]) and aggressive behavior symptoms (*B* = .47, *SE* = .19, *p* = .016, 95 % CI = [.09–.85]), but not distress symptoms (*B* = .85, *SE* = .58, *p* = .14, 95 % CI = [−.30–2.01]). Significant pathways between higher PTE exposure and reduced HRV mediated by increased age were identified in each model, demonstrating the mediating effect of age on the relationship between trauma exposure and HRV reductions. However, there was no mediating effect of HRV in the relationship between PTE exposure and mental health outcomes of PTSD, distress, or aggressive behavior. Whereas the age and HRV-mediated pathway predicting PTSD symptoms was close to significance (indirect bootstrapped *B* = .006, *SE* = .005, 95 % CI = [−.0001–.0196]), the pathways for distress (indirect bootstrapped *B* = .06, *SE* = .07, 95 % CI = [−.02–.26]) and aggressive behavior (indirect bootstrapped *B* = .02, *SE* = .03, 95 % CI = [−.02–.10]) showed no such trend. Sex did not exert a significant effect on any pathway or effects.

#### Full mediation models

**Fig. 2:**
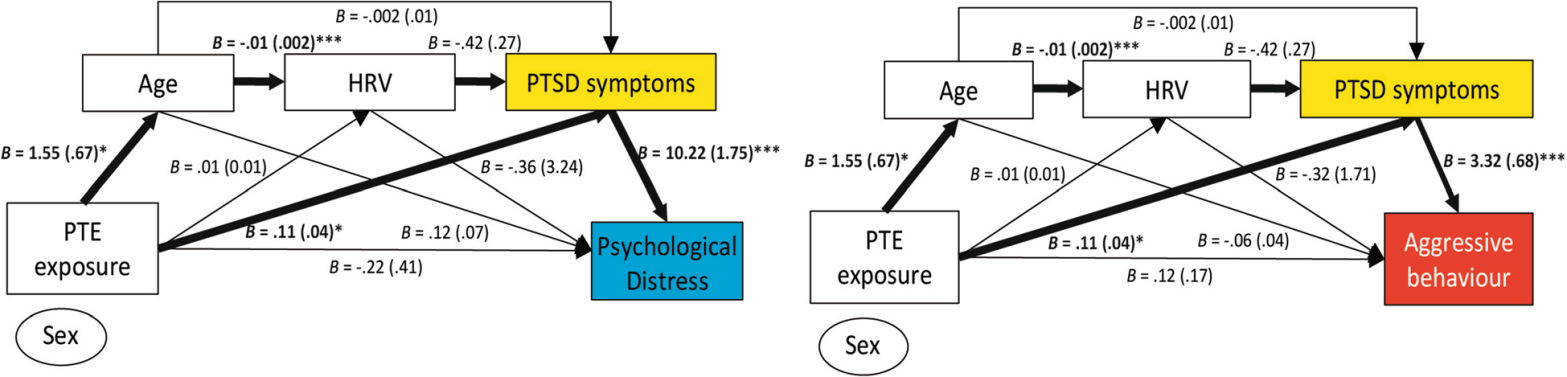
Mediation models testing the association between PTE exposure and symptoms of psychological distress (*left*) and aggressive behavior (*right*) via indirect age, HRV and PTSD symptom serial pathways. *Thick lines* indicate significant direct or indirect pathways. Effects sizes (standard errors) are presented for each path; * *p* < .05; ** *p* < .01; *** *p* < .001. The covariate of sex was not significantly associated with any of the variables in the model

Figure 2 presents the full mediation models examining the association between PTE exposure and symptoms of distress and aggression respectively, mediated serially by age, HRV, and PTSD symptoms. Overall, the total effect of PTE exposure on each outcome measure was significant: PTSD symptoms (*B* = .11, *SE* = .04, 95 % CI = [.03–.18], *R*^2^ = .18); distress (*B* = .1.02, *SE* = .50, 95 % CI = [.03–2.01], *R*^2^ = .09); aggressive behavior (*B* = .38, *SE* = .18, 95 % CI = [.02–.74], *R*^2^ = .07).

Direct pathways between PTE exposure and distress (*B* = −.22, *SE* = .41, *p* = .59, 95 % CI = [−1.05–.60])) and aggressive behavior (*B* = .12, *SE* = .17, *p* = .49, 95 % CI = [−.22–.46])) were not significant. However, two significant mediation effects were found in both models. A significant indirect serial pathway between PTE exposure and greater psychological distress symptoms was mediated by the sequence of older age, lower resting HRV, and elevated PTSD symptoms (indirect bootstrapped *B* = .06, *SE* = .05, 95 % CI = [.001–.217]). A comparable indirect serial pathway was found for the association between PTE exposure and aggressive behavior, with the same sequence of older age, lower HRV, and elevated PTSD symptoms also mediating the relationship (indirect bootstrapped *B* = .02, *SE* = .02, 95 % CI = [.0003–.069]).

The second mediating effect demonstrated that greater PTE exposure was also associated with both elevated distress symptoms (indirect bootstrapped *B* = 1.08, *SE* = .45, 95 % CI = [.30–2.07]) and aggressive behavior (indirect bootstrapped *B* = .35, *SE* = .15, 95 % CI = [.12–.72]) mediated via PTSD symptom severity. Again, sex did not exert a significant effect in any model.

In the first alternative mediation model tested (a), a direct relationship between PTE exposure and PTSD symptoms (c΄ = .07, *SE* = .03, 95 % CI = [.01–.12]) and aggressive behavior (c΄ = .42, *SE* = .20, 95 % CI = [.03–.82]) was found; no mediating effects were observed. The second alternative model (b) found an association between greater PTE exposure and heightened PTSD symptoms mediated by aggressive behavior (indirect bootstrapped *B* = .03, *SE* = .01, 95 % CI = [.01–.06], but no effects involving age or HRV were observed.

## Discussion

This study is the first to report on heart rate and heart rate variability (HRV) data collected amongst a highly trauma-exposed community sample in a post-conflict setting. Resting HRV was lower in the case group comprising PTSD, MDE and IED diagnoses compared to the non-case group, yielding a moderate effect size (*d* = 0.5). A significant association between reduced HRV and older age was observed, accordant with the extant literature that HRV diminishes naturally with age [24, 26, 27]. Resting heart rate, by contrast, did not significantly vary between cases and noncases. The mediation models found that age-related HRV reductions were associated with increases in PTSD symptoms, a relationship that significantly accounted for the dose-response relationship between PTE exposure and symptoms of psychological distress and aggressive behavior. This finding supports a more complex model in which emotion dysregulation, reflected in lowered resting HRV [11], underpins an increased sensitivity to the development and maintenance of posttraumatic stress psychopathology following cumulative PTE exposure and the onset of PTSD symptoms, while also accounting for age effects. While recognizing this mediation effect is based on cross-sectional data, the convergence between the group differences and serial modelling highlight the possible contribution of age-related lowered HRV to the maintenance of mental health symptoms in conflict-affected groups.

The finding of lower resting HRV in the case group is consistent with the body of work that has linked parasympathetic disturbances to a wide range of mental disorders, including PTSD, depression and aggressive anger [18–20, 22]. The case group appears to be characterized by reduced cardiac vagal capacity, which prior research has shown to be related to diminished emotion regulation [13, 15] – a clinical feature of both PTSD and IED. Such reduced cardiac vagal capacity is also related to poor adaptive responding to both stressful and social situations [10, 11, 13, 17]. The finding of lowered resting HRV in the case group in this Timorese sample is a novel aspect of our study, suggesting that disruption to the vagal mechanism may be a universal biological marker of psychopathology regardless of setting or culture [48].

The mediation effects suggest that the relationship between cumulative PTEs and symptoms of distress and aggressive behavior is influenced by a sequence of age-related HRV reductions *and* increased PTSD symptoms. Notably, HRV was not a significant mediator in the initial baseline models testing for direct associations between PTE exposure and the mental health outcomes. In respect to the first part of the model, the relationship between PTE exposure and HRV does not appear to follow a linear relationship, with age appearing to be an important contributing factor to the mediating role of HRV. Cumulative trauma exposure may weaken vagal tone over time [14], compounding the normal reduction in HRV with age [25]. Chronic reductions in HRV appears to also underpin risk for future ill-health, the development of serious medical conditions and mortality [17, 49], a result of impaired vagal inhibition leading to dysregulation within the anti-inflammatory pathway [50]. The present study indicates that age-related HRV reductions may bear an important relationship to chronic mental health problems in this Timorese sample, who have been exposed to decades of conflict, human rights and deprivation-related PTEs.

In respect to the second part of the model, the findings accord with previous longitudinal studies demonstrating that PTSD symptoms following PTE exposure precedes and influences the subsequent development of depression, anxiety and other disorders amongst combat veterans [7], survivors of abuse [9] and those exposed to a range of other traumas in the general population [8]. Notably, PTSD symptoms were also shown to mediate the association between PTE exposure and psychological distress and aggression, independent of age and HRV. However, the inclusion of age and resting HRV in the expanded model suggests a complex relationship in which parasympathetic disturbances may represent a psychophysiological pathway linking PTE exposure, PTSD symptoms and psychological distress/aggressive behavior - both prominent mental health issues in Timor Leste [4, 5]. Mapping these pathways may assist in advancing understanding of the mechanisms of psychological adaptation of populations in post-conflict countries in general.

Limitations of the study include the non-representative nature of the sample which was deliberately weighted towards including a substantial case group. The higher prevalence of IED is broadly consistent with Timorese community prevalence rates relative to PTSD and depression in general [5]. The advantage of the stratification procedure was that sufficient numbers with the key diagnoses of PTSD, IED and MDE were included, which together with the non-case control group, provided adequate power and variability to conduct mediation pathway analyses. Considering the smaller numbers in each of the diagnostic categories, disorder or comorbidity specific effects cannot be ruled out and will need to be examined in larger samples. Comorbid physical medical conditions that are known to impact on indices of HR were not measured in the present study because of low levels of accuracy in assessing physical health in this setting based on participant reports or health records. RMSSD was selected as the index of HRV in the present analyses, but there are other indices that may also prove useful to index aspects of vagal responsivity such as respiratory sinus arrhythmia – a measure that we were not able to assess due to contextual constraints and the use of the Polar RS800CX equipment. However, we also highlight evidence that RMSSD is a stable index of HRV over short recordings [46], and that the measurement of respiration can interfere with the association between HRV and vagal tone [51]. The cross-sectional nature of the study precludes drawing any firm inferences concerning causality; only longitudinal inquiries can assess whether changes in HRV are a cause or consequence of psychopathology following trauma exposure.

## Conclusions

In general, our findings add to the growing body of evidence of an association between lowered HRV and psychopathology [17], suggesting that this relationship may extend to a conflict-affected population in a transcultural setting. In particular, our findings allow a refinement of a model linking PTEs to psychological distress and aggressive behavior, an association that was found to be mediated by age-related reduced HRV and PTSD symptoms. Further research is needed to determine the generalizability of this finding to other populations, and to establish the chronological sequencing of these relationships between environmental stress, biological mechanisms and psychological reactions.

### Ethics

Participants provided consent to take part in the study according the ethics approval provided by the University of New South Wales (UNSW) Human Research Ethics Committee. This included consent to publish group analyses in peer reviewed scientific journals.

### Availability of data and materials

Non Applicable.
